# Views of City, County, and State Policy Makers About Childhood Obesity in New York State, 2010–2011

**DOI:** 10.5888/pcd10.130164

**Published:** 2013-11-21

**Authors:** Rebecca Robbins, Jeff Niederdeppe, Helen Lundell, Jamie Meyerson

**Affiliations:** Author Affiliations: Rebecca Robbins, Jamie Meyerson, Cornell University, Ithaca, New York; Helen Lundell, The Hartman Group, Inc, Seattle, Washington.

## Abstract

**Introduction:**

No single solution exists to reduce rates of childhood obesity in the United States, but public policy action is essential. A greater understanding of policy maker views on childhood obesity would provide insight into ways that public health advocates can overcome barriers to propose, enact, and implement obesity prevention policies.

**Methods:**

We conducted 48 in-depth, qualitative interviews with town/city, county, and state policy makers in the state of New York from December 14, 2010, through June 10, 2011. We used a semistructured interview protocol to solicit policy maker views on the causes of, solutions to, and responsibility for addressing the issue of childhood obesity.

**Results:**

Most policy makers considered the issue of childhood obesity to be of high importance. Respondents cited changes to family structures as a major cause of childhood obesity, followed by changes in the external environment and among children themselves. Respondents offered varied solutions for childhood obesity, with the most common type of solution being outside of the respondent’s sphere of policy influence. Policy makers cited the need for joint responsibility among parents, government, schools, and the food industry to address childhood obesity.

**Conclusion:**

Beliefs of many policy makers about childhood obesity are similar to those of the general public. Findings highlight the need for future research to inform the development of communication strategies to promote policy action among those with authority to pass and implement it.

## Introduction

Changes in physical, social, economic, information, and policy environments during the past 30 years have contributed to large increases in childhood obesity rates in the United States (1,2). No single solution will be sufficient to offset these trends — large reductions in childhood obesity rates require action from multiple stakeholders, and public policy action is essential (3–16).

There is growing support among health advocates and officials for policy attention to childhood obesity across all levels of government (17,18). Legislators are beginning to heed that call; policies addressing childhood obesity were proposed in all 50 states between 2003 and 2005 (19). Recent policy attention has focused on areas such as food marketing to children (20,21) and taxes or portion size bans to reduce sugar-sweetened beverage (SSB) consumption (4). Many of these policies have encountered challenges in gaining public and policy maker support (5,6).

Research has identified situational factors that hinder obesity-related policy enactment (including powerful industry lobbyists, lack of bipartisan support, and regulatory ambiguity) (6–8) and factors that enable policy enactment (including bipartisan sponsorship, national media exposure, and federal resource allocation to address the issue) (7,9,19). However, research on policy makers’ personal views about childhood obesity is scarce.

A greater understanding of policy maker views on childhood obesity would provide insight into ways that public health advocates can overcome barriers to propose, enact, and implement obesity prevention policies. We examined policy maker beliefs about the importance of, causes of, and possible solutions to childhood obesity. 

## Methods

We conducted 48 in-depth, qualitative interviews with town/city, county, and state policy makers in New York State from December 14, 2010, through June 10, 2011. We asked 5 research questions: 1) Where does childhood obesity fall among other policy maker priorities?, 2) What do policy makers see as the causes of the rise in childhood obesity over time?, 3) Whom do policy makers see as responsible for addressing childhood obesity?, 4) What do policy makers see as feasible, effective, and appropriate policy solutions for childhood obesity?, and 5) Do policy maker views differ by level of government, role in government, or political party? Interviews averaged 45 minutes and were audiotaped with consent.

We used public records to obtain contact information for town supervisors and city mayors of municipalities with more than 10,000 residents (20 contacted, 11 interviewed), county health officials (44 contacted, 21 interviewed), county executives (34 contacted, 4 interviewed), and state legislators (State Assembly and Senate: 33 contacted, 12 interviewed) who lived within 2 hours of Cornell University (a 90-mile radius). We contacted respondents by e-mail and followed up by telephone. When schedules permitted, we conducted interviews in the policy maker’s office, although more than half of the interviews (n = 33) were conducted via telephone. We asked telephone interviewees to have e-mail access at the time of the conversation so we could send them messages for discussion at the appropriate time; in a few cases we sent these before the interview. We obtained informed consent for conducting and recording the interview by e-mailing participants a consent form. All respondents returned it in person or via e-mail or fax before the interview. The study was approved by Cornell University’s institutional review board.

The second (J. N.), third (H. L.), and fourth (J. M.) authors conducted the interviews using a semistructured interview protocol, although interviewers had freedom to ask additional follow-up questions at their discretion. We trained interviewers by pilot testing the interview protocol on (nonauthor) members of the research team. Each interviewer had experience with qualitative data collection.

### Interview guide

The semistructured interview began with questions about the importance of childhood obesity (research question [RQ] 1; Appendix). Respondents then viewed a graph (www.cdc.gov/nchs/data/hestat/overweight/overweight_child_03.htm) depicting changes in rates of childhood obesity in the United States between 1963 and 2004 and were asked to identify causes of the increase in childhood obesity (RQ2) and their beliefs about responsibility for addressing this issue (RQ3). Respondents then viewed a second graph, this time presenting data on rates of childhood obesity in New York by household income (n = 27) or race/ethnicity (n = 21). Each participant saw only 1 graph; we typically alternated the content of the images but chose to present the income image in a few cases where the demographic composition of the city/town or county was racially and ethnically homogenous. We concluded the interview by asking respondents for their views on what should be done to reduce rates of childhood obesity in New York (for state legislators) or the respondent’s county or town (RQ4).

### Analysis

The interviews were transcribed by a professional transcription service and then coded line-by-line by the study team in ATLAS/tio version 4.2 (Scientific Software Development, Berlin, Germany). The lead author (R. R.) conducted the analysis, organizing comments into major themes and subcategories. Codebook development was informed by literature on public beliefs about childhood obesity but was also open to inductive categories emerging from the data. The analysis proceeded in waves; themes that emerged were routinely reported to the other members of the research team for review, feedback, and codebook refinement. The second author (J. N.) reviewed all categories and confirmed agreement with the coding throughout the process. When disagreements or confusion in the interpretation of respondent comments arose, researchers returned to the data for further analysis and came to consensus. To address RQ5, we looked carefully for distinctions between responses by levels of government (town/city, county, state), roles (health or broader public official), and political party (Republican or Democrat). We saw few patterns of difference across these distinctions and thus do not report results separately by these groups except in rare cases (solutions) where differences were clear. We planned to analyze comments on perceptions of disparities in childhood obesity (following graphs depicting social group differences), but most of these conversations focused on broader factors that influence childhood obesity in the United States, rather than on disparities per se. We conducted member checks by sending the manuscript to respondents for feedback, but no participants requested any modifications.

## Results

Forty percent of respondents were female. The sample was a mix of elected and appointed officials: 12 were state legislators, 11 were city mayors or town supervisors, 4 were county executives, and 21 were county health directors or commissioners. Elected officials typically disclosed their political party affiliation publicly (obtained via a Web search), but appointed officials generally did not. Just under one-quarter of respondents (n = 11) were Democrats, approximately 30% were Republicans (n = 14), and approximately half did not publicly disclose their party affiliation (n = 23).

### Importance of childhood obesity (RQ1)

Despite the diversity of political offices held by respondents (and associated responsibilities that could prioritize certain issues over others), most stated that childhood obesity is a high priority issue. Respondents with this view believed that there was a high level of national attention to the issue and potential for local and societal impact by addressing it. For example, one state legislator explained the importance of addressing childhood obesity in terms of its far-reaching negative consequences (Table).

**Table:** Illustrative Quotes, Qualitative Interview of City, County, and State Policy Makers About Childhood Obesity, New York State, 2010–2011

Category	Subcategory	Quote	Office
Importance of childhood obesity (RQ1)	High importance	“The economics of this aren’t just on the health care side, it’s in terms of the potential for these children to become productive members of the workforce and help us solve these problems down the road.”	State
	Low importance	“Well, as far as my position as mayor when I’m working with city issues, [childhood obesity] is very low.”	Town

Causes of childhood obesity (RQ2)	Parental/family-related causes	“I think also tied to it though is a cultural change, where the family situation is such that both the parents or you know, we have single-parent homes, you know, the parent is out working to provide for the family, so the sit down at home over, you know, a full-course meal, that’s balanced, that’s becoming more difficult for families to achieve.”	State
		“I think parents are somewhat responsible because they need to be more in-tune to providing good and nutritional things to our families. I think we need to limit the amount of access to [television] that we have our kids watch.”	County: Health
	External causes	“People are on computers more, kids are playing video games — we never had video games growing up.”	State
	Internal causes	“Kids [are] staying in and playing video games instead of going outside and playing, and instead of walking over to see a friend you can just text them and talk to them on the Internet, on your phone, on your iTouch.”	County: Executive

Responsibility to address childhood obesity (RQ3)	Joint responsibility	“It’s everybody’s responsibility. I mean, we’ve got physicians who are fat, so how can they tell somebody to lose weight, right? I mean, it’s hard. It’s really hard to maintain your weight. So, it’s everybody’s responsibility.”	State
		“It has to be a joint effort to look at the obesity rates and to try to reduce the intake that we have and it’s just not one individual or one agency.”	County: Health
	Parents	“Because they are really in charge of . . . number one, modeling good behavior, number two, ensuring that the children are eating . . . the right amount of calories and ensuring that the kids are getting the right amount of physical activity. They’re responsible for ensuring that kids unplug, you know a number of the stuff that they do that promotes a sedentary lifestyle or recreation. So, the parents first.”	County: Health
		“So that brings in the parent. You know . . . and like I said, I’m not blaming this . . . they have the kids the most. But the parents have the other . . . the most. I believe that there are a lot of them that aren’t paying attention to this activity.”	County: Health
	Government	“I think it has to be federal because otherwise, you’ve got this system where one state has one thing going and another state has . . . but if you look at the states that have the highest levels of obesity, quite frankly, it looks like the states that have the least regulatory function.”	Town
	Schools	“I think probably schools have a lot to do that with school activities, school education . . . because children can often bring things home from the school in order to help a household understand, you know, better nutrition or better activities.”	Town
	Food industry	“And industry, frankly. I mean, I don’t want to leave out the food industry. I think we have to ask them to step up to the plate as well and . . . stop giving us as much junk as they’re giving us, you know?”	State

Solutions for childhood obesity (RQ4)	Hypothetical solutions	“I’d love to see something in place for schools, like a club for the overweight, or free clinics where individuals are not forced to join.”	County: Executive
		“Create resources and tool kits that can help parents, caregivers and school officials with this problem . . . so we connect them to the resources like online BMI calculators.”	Town
	Existing solutions	“We’re working with one school, elementary school, that for a child’s birthday celebration, rather than the parent bringing in cake and cupcakes and ice cream and all of that, they’re, we’re piloting that the child’s, on the day the child’s birthday is celebrated they get an extra recess to play. Be at play outside, play inside, depending on the weather, for more activity. Again, to promote that activity, the physical activity aspect rather than associating a celebration always with food. Particularly unhealthy food.”	County: Health
	Proposed solutions	“We have also in the school district situation some proposed legislation in connection with whether or not the soft drinks should be in vending machines in school districts and allowed to advertise things of that nature.”	State

Abbreviation: RQ, research question; BMI, body mass index.

Policy makers at the county level were more likely to regard other health concerns as more pressing. The handful of respondents who thought that childhood obesity was of moderate importance or simply not a priority believed that addressing this issue is someone else’s job (Table). Other reasons cited for childhood obesity being of moderate or low importance included competing priorities, political factors (like a lack of bipartisan support), or limited resources.

### Causes of childhood obesity (RQ2)

Attribution theory holds that people make sense of their environment by assigning responsibility for events or dispositions (eg, becoming obese) (10–12). These judgments are often classified as internal (controllable by the individual) or external (outside of a person’s control) (13). We classified responses about causes of childhood obesity into 3 categories: 1) parental or family-related, 2) external (eg, people are embedded in a toxic food environment), and 3) internal (eg, people make bad decisions about diet and exercise).

The most frequently cited cause of childhood obesity had to do with parental and family-related factors, because a child’s ability to make sound health decisions is legally and developmentally bound by parental decisions and the family environment. Comments in this category cited the changing nature of the family, such as increases in the number of dual-working parents, which were cited as contributing to undesirable effects on children’s diet (eg, fewer home cooked meals, more consumption of fast or convenience foods) and physical activity (no one at home encouraging kids to play outside). A handful of respondents cited as a primary cause poor parenting, such as parents placing their kids in front of the television and modeling unhealthful behaviors (Table).

The second most commonly cited causes of childhood obesity were external factors that shape dietary behavior. Respondents discussed varied factors including limited access to healthful food (high cost, limited availability) and easy access to unhealthful food (low cost, readily available). A smaller number of respondents mentioned direct-to-child marketing and poor nutrition in school meals.

Fewer respondents cited lack of opportunities for physical activity. These comments focused primarily on large-scale societal changes (eg, culture of playing inside vs outside) that have created unhealthy social norms for children’s behavior. The availability of video games was emphasized by one respondent, illustrating a sense that there have been broader social changes rather than dispositional changes in parents or children themselves (Table).

The least-often cited causes of childhood obesity had to do with factors internal to children themselves. These causes largely focused on children’s preference for unhealthful food options (fast or convenience foods, high-calorie foods, and SSBs). Some respondents also mentioned internal causes of childhood obesity related to physical activity. These comments suggested that children prefer sedentary behaviors, such as watching television, playing video games, or using the Internet, to outdoor play and other forms of physical activity (Table). This view acknowledges technological changes that make it easier to socialize without having to go outside, but it also emphasizes the role played by preferences among children themselves.

### Responsibility to address childhood obesity (RQ3)

Several sources of responsibility, including parents, government, schools, and the food industry, were mentioned in response to questions about who should address the problem of childhood obesity. Most of the sample explicitly described the idea of joint responsibility (referring to members of more than one group, often specifically listed) as crucial for finding a solution to childhood obesity. Parents were the most commonly cited source of responsibility. Approximately half the comments about parents cited the need for developing good habits during the childhood years (Table). Respondents also cited the need for parents to model good behavior and not shirk responsibility for being a good parent.

The next most commonly cited source of responsibility was government, although no particular level of government at which these efforts should be focused was mentioned. Comments had to do with the need for government-funded obesity education (in general or specifically targeting parents) or food industry regulation (addressing the use of unhealthful ingredients, product labeling, and child-targeted marketing). The third most-cited source of responsibility was schools. Respondents described inadequate education about health in the school system, poor nutritional offerings in the cafeteria, and declining physical education programs due to funding or other constraints.

### Solutions for childhood obesity (RQ4)

Comments about solutions for childhood obesity occurred at several points during the interview: following questions about responsibility for addressing childhood obesity, differences between groups, and ideas about what could be done to address the problem. We coded responses into 3 categories: hypothetical solutions, existing solutions, and proposed solutions.

Hypothetical solutions were most common (approximately half of the comments about solutions) and were coded as such for their use of wishful (eg, “I’d love to see”) rather than concrete language (eg, “We should do this in our roles as policy makers”). The most often-cited (about one-third of participants) hypothetical solution was increased general education about diet and exercise, without specific mention of where or how to provide it. Many other, more specific hypothetical solutions were characterized by a focus on individual skill building for children. These ideas ranged from curricula to teach and promote physical activities and healthful eating in school (Table), developing popular television programing with a focus on healthful diet and exercise, and celebrity endorsements of healthful eating and active living. Less common, voiced by a handful of respondents, were ideas about structural changes that could reduce rates of childhood obesity. A few respondents discussed the importance of regulating the food stamp program to prohibit purchases of junk food, and several others mentioned regulating school nutrition programs. Others cited the need to conduct a needs assessment (Table), and some discussed the value of increasing the minimum wage. A few respondents mentioned increasing workplace wellness efforts, regulating calorie labeling, providing radio-based education, creating parks and playgrounds, and increasing access to local foods. County health and state policy makers contributed nearly all of the hypothetical solutions (one of only a few clear patterns of difference observed in the study, addressing RQ5).

Existing solutions were the second most frequently cited (just under half of total solutions) and had to do with solutions already in practice that policy makers had implemented personally or oversaw among their subordinates. Existing solutions were more specific and concrete than the hypothetical ones (Table); most frequently cited (by 6 to 10 participants) included providing education programs to promote healthful eating in general, programs to enhance school nutrition, and programs to promote physical activity; increasing the number of or access to parks and playgrounds; allocating funding to childhood obesity prevention; and developing community gardens. Less frequent but still prominent (2–5 participants) were providing farmers markets, creating trails and sidewalks, and increasing access to local foods.

A few policy makers described specific solutions that had not yet been implemented but were more specific than the hypothetical ones discussed. Most described programs that were at some point in the legislative pipeline, including monitoring body mass index in schools, developing parks and playgrounds, and implementing policies (like taxes or school restrictions) to reduce consumption of SSBs (Table).

When discussing solutions, several policy makers acknowledged obstacles to policy action at their level of government. The most common obstacle was limited funding. A handful of respondents mentioned the influence of powerful lobbyists, challenges of reaching people with low income, lack of public support for childhood obesity initiatives, and other priorities drawing attention away from childhood obesity.

## Discussion

This study describes beliefs about childhood obesity among policy makers at multiple levels of government in New York State. Results are encouraging for public health advocates who seek increased policy attention to childhood obesity. Most policy makers, across varying levels of government, saw childhood obesity as an issue worthy of concern and attention and as a problem with many causes that needs to be addressed by multiple stakeholders at several levels. Policy maker perspectives on childhood obesity and policy were largely consistent with previous research among the general public (12,22), which suggests that public opinion research has the potential to inform the development of communication strategies to promote policy action among those with authority to pass and implement it. Knowledge of factors that policy makers see as central to the issue can inform efforts to frame the topic in ways that enhance policy support and catalyze efforts to advocate for their passage.

However, findings indicate challenges to future efforts to reduce childhood obesity rates through public policy that may be best addressed by targeting policy makers at specific levels of government. Some policy makers saw the issue of childhood obesity as beyond the scope of their office or position, better addressed at other levels of government or nongovernmental organizations. Solutions to the broader problem of childhood obesity were discussed largely in hypothetical terms, offering novel and useful ideas but perhaps without a high likelihood of immediate action or implementation. Lack of funding for obesity-related initiatives was a common theme across topics of discussion, and many of the recommended types of programs (large-scale education through schools or the media) require substantial resources. Few respondents mentioned that broader policy making could consider health effects of policies that are not explicitly health-oriented but may have implications for childhood obesity (eg, transportation infrastructure, neighborhood safety) (18). Research to identify policies that provide both economic and public health benefit, and communication strategies to promote them, seem appropriate in response to concerns about limited budgets for childhood obesity prevention.

This study is limited in its reliance on a convenience sample of policy makers living in a specified geographic area at a certain time. The limited sample size and qualitative data limit the degree to which these findings can be generalized to other states or issues. Policy makers with heightened interest in childhood obesity prevention may have been more likely to agree to participate, biasing results toward greater support for policy to prevent childhood obesity. Future work should explore policy maker views about childhood obesity in different contexts, including other areas of the country and broader levels of government such as large cities, federal legislators, and major advocacy organizations.

## Appendix. Semistructured Interview Guide

**Concern About the Issue of Childhood Obesity**

1. [Mayors/senators/representatives] have to deal with many issues of concern to their constituents. Where does addressing rates of childhood obesity in [your city/New York] fall among all of the other priorities you have to consider? Why do you think this is the case?

**Explanations for Increases in Childhood Obesity**

2. Let’s continue by taking a look at some statistical data compiled by the CDC on the growth of rates of childhood obesity in the United States.

(SHOW IMAGE SHOWING GROWTH IN OBESITY RATES)

2a. What comes to your mind as you are looking at this chart?

3. Why do you think rates of obesity have changed over the past 25 years?

4. Who do you think is responsible for reducing rates of childhood obesity in New York?

**Disparities in Childhood Obesity**

5. Now let’s take a look at a second chart showing differences in rates of childhood obesity in the United States by [combination of RACE/INCOME/UPSTATE or DOWNSTATE].

(SHOW CHART SHOWING DIFFERENCES IN OBESITY RATES)

5a. What comes to your mind as you are looking at this chart?

5b. Why do you think rates of obesity differ between these groups?

6. Who do you think is responsible for reducing differences in rates of childhood obesity [by RACE/INCOME/GENDER]?

**Solutions to Childhood Obesity**

7. In your role as a [MAYOR/STATE LEGISLATOR], what do you think could be done, if anything, to reduce rates of childhood obesity in [STATE/CITY]? Why?

7a. PROBE: What are the barriers to doing this?

7b. PROBE: What else do you think could be done?

8. Would there be any advantages for your [CITY/STATE] if you were able to reduce rates of childhood obesity in [CITY/STATE]? What might these be?

**Closing Thoughts**

9. Is there anything related to childhood obesity that we didn’t talk about today that you’d like to have the opportunity to share?
